# Evaluation of enzymatic activity, hemolytic safety, and antibiotic susceptibility of *Bacillus* spp. isolated from giant freshwater prawn *(Macrobrachium rosenbergii*) farms in Kalasin Province, Thailand

**DOI:** 10.14202/vetworld.2025.3622-3630

**Published:** 2025-11-29

**Authors:** Keeravit Petjul, Nattapon Kan-a-roon, Prasit Khunsanit, Urai Kollboon, Tanaphoom Boonmee

**Affiliations:** 1 Department of Interdisciplinary Agriculture, Faculty of Agricultural Technology, Kalasin University, Mueang District, Kalasin Province, 46000 Thailand; 2 Department of Fisheries Technology, Faculty of Agricultural Technology, Kalasin University, Mueang District, Kalasin Province, 46000 Thailand

**Keywords:** antibiotic susceptibility, *Bacillus infantis*, *Bacillus pseudomycoides*, *Bacillus safensis*, enzyme activity, hemolytic safety, *Macrobrachium rosenbergii*, probiotics

## Abstract

**Background and Aim:**

The giant freshwater prawn (*Macrobrachium rosenbergii*) is an economically valuable aquaculture species, yet its production often faces challenges related to poor growth and disease outbreaks caused by intensive farming practices and excessive antibiotic use. Probiotics offer a sustainable alternative for improving growth, immunity, and pond health, but their efficacy and safety are highly strain specific. This study aimed to evaluate the probiotic potential of *Bacillus pseudomycoides*, *Bacillus safensis*, and *Bacillus infantis* isolated from prawn farms in Kalasin Province, Thailand, focusing on digestive enzyme activities, hemolytic safety, and antibiotic susceptibility.

**Materials and Methods:**

Three *Bacillus* strains previously identified by 16S ribosomal RNA sequencing were characterized using standard *in vitro* assays. Amylase, protease, and lipase activities were assessed using the halo/colony (H/C) ratio and enzyme unit measurements. Hemolytic patterns were examined on Columbia agar supplemented with 5% sheep blood, and antibiotic susceptibility was tested by the disk diffusion method against seven antibiotics following Clinical and Laboratory Standards Institute (2024) guidelines. All assays were performed in triplicate, and data were analyzed using one-way analysis of variance (p < 0.05).

**Results:**

All strains exhibited strong amylolytic (H/C ratio 2.05–2.27) and proteolytic (H/C ratio 1.75–1.96) activities, while lipase activity was undetectable. Hemolysis testing revealed γ-hemolysis for all strains, confirming non-hemolytic and non-pathogenic properties. Antibiotic susceptibility profiles indicated broad sensitivity to penicillin G, tetracycline, streptomycin, erythromycin, and chloramphenicol. Moderate susceptibility to vancomycin was observed in *B. pseudomycoides* and *B. safensis*, while *B. infantis* remained fully susceptible. The results suggest a strong digestive enzyme potential and an acceptable safety profile among all isolates.

**Conclusion:**

The evaluated *Bacillus* strains exhibit favorable probiotic attributes, including high amylase and protease activity, non-hemolytic safety, and broad antibiotic susceptibility, which support their suitability for probiotic application in *M. rosenbergii* culture. These native isolates may serve as sustainable, locally adapted alternatives to antibiotics, contributing to improved feed efficiency, growth, and disease resistance in freshwater prawn aquaculture. Further *in vivo* validation and genomic safety analyses are recommended to confirm their efficacy under commercial conditions.

## INTRODUCTION

The giant freshwater prawn (*Macrobrachium rosenbergii* de Man) is one of the most economically important freshwater species, valued for its high market price and consumer demand. In 2017, Thailand’s aquaculture sector produced approximately 16,693 tons of giant freshwater prawns, worth about 5,133 million baht. Most local farmers adopt high-density, low-cost farming practices, which, although economically attractive, often lead to production challenges such as low juvenile survival rates, slow growth, stunted development, and increased susceptibility to viral and bacterial diseases. These recurring issues frequently result in reduced yields, driving farmers to rely heavily on antibiotics and chemical treatments.

The indiscriminate and prolonged use of antibiotics in shrimp and prawn aquaculture has become a global concern due to its role in environmental pollution, the development of antibiotic resistance, and the contamination of aquatic ecosystems [1, 2]. In Thailand, aquaculture zones, particularly those cultivating giant freshwater prawns, have a long history of chemical use. Such practices contribute to the accumulation of chemical residues, including fertilizers and prohibited substances, in pond sediments and nearby water bodies. Consequently, farmers are often required to rehabilitate or modify ponds between production cycles to restore water quality.

Yang Talat District in Kalasin Province is a major prawn-farming area that benefits from abundant water supplied by the Lam Pao Dam and a well-developed irrigation network. However, despite these favorable conditions, the region continues to face persistent problems related to chemical residues and disease outbreaks. The overuse of chemicals not only increases production costs but also leaves harmful residues in prawns and pond water, aggravating environmental contamination and threatening the Lam Pao Dam ecosystem [3].

To address these challenges, the application of probiotics in aquaculture has emerged as a promising, eco-friendly approach for enhancing growth performance, disease resistance, and water quality [4, 5]. Recent trends in organic shrimp farming have shown that probiotic bacteria can effectively suppress pathogenic microbes and improve animal health, particularly in black tiger shrimp (*Penaeus monodon*) [6]. A variety of microorganisms, including bacteria, yeasts, and fungi, can act as probiotics, with lactic acid bacteria (e.g., *LactoBacillus bulgaricus*, *LactoBacillus acidophilus*, *LactoBacillus casei*, *Enterococcus*) and yeasts (*Saccharomyces cerevisiae*, *Saccharomyces boulardii*) being the most widely used [7, 8].

Studies have also demonstrated the benefits of *Bacillus* species in aquaculture systems. For example, the application of *Bacillus subtilis* and *Bacillus licheniformis* in grass carp (*Ctenopharyngodon idellus*) culture enhanced antioxidant capacity and immune response [9]. Similarly, probiotic *LactoBacillus* strains isolated from coastal soils have shown potential to reduce ammonia and nitrogen levels while improving shrimp growth performance [10]. Building on such findings, the present study aimed to evaluate the probiotic potential of *Bacillus* spp. strains isolated from giant freshwater prawn ponds in Kalasin Province, Thailand. The objective was to assess their enzymatic activity, safety, and antibiotic susceptibility to determine their suitability for development as locally adapted probiotics to enhance prawn production and reduce reliance on chemical inputs.

In addition to their digestive enzyme production and safety profile, probiotic *Bacillus* species have been reported by Sam-On *et al*. [11] and Proespraiwong *et al*. [12] to promote growth and inhibit major pathogens such as *Aeromonas hydrophila* and *Vibrio* spp. These properties highlight the potential of *Bacillus*-based probiotics to enhance immune response, improve feed utilization, and increase disease resistance in *M. rosenbergii*, thereby contributing to a more sustainable aquaculture system.

Although several studies have demonstrated the beneficial effects of *Bacillus* species in aquaculture, most previous work has focused on commonly used strains such as *B. subtilis* and *B. licheniformis*. For instance, the use of these species in grass carp *(C. idellus*) significantly improved immune responses and antioxidant activity, supporting their role as potent probiotics in aquatic species [9]. However, limited information exists on the probiotic potential of other *Bacillus* species that are naturally adapted to local pond environments, particularly those associated with giant freshwater prawn *(M. rosenbergii*) culture systems. Furthermore, the majority of prior research has emphasized growth performance and general health outcomes, with relatively few studies systematically assessing both safety and functional enzyme activity of locally isolated *Bacillus* strains. Recent findings have shown that *B. licheniformis* supplementation enhances growth, survival rate, and immunity in *M. rosenbergii* [13], yet such research remains largely confined to a narrow range of commercial species. This knowledge gap highlights the need to explore native *Bacillus* isolates that may possess unique probiotic properties, environmental resilience, and compatibility with tropical pond conditions typical of Thailand’s aquaculture systems. A more comprehensive understanding of these locally derived strains is crucial to developing sustainable probiotic formulations that can reduce antibiotic dependence and improve farm productivity.

This study aimed to evaluate the probiotic potential of native *Bacillus* spp. strains isolated from giant freshwater prawn farms in Kalasin Province, Thailand. Specifically, the objectives were to (1) determine their digestive enzyme activities (amylase, protease, and lipase), (2) assess their hemolytic safety to ensure non-pathogenic characteristics, and (3) examine their antibiotic susceptibility profiles to confirm biosafety for aquaculture application. By characterizing these locally adapted *Bacillus pseudomycoides*, *Bacillus safensis*, and *Bacillus infantis* isolates, this study seeks to provide foundational evidence supporting their potential use as eco-friendly probiotics to enhance growth performance, digestive efficiency, and disease resistance in *M. rosenbergii* culture while promoting sustainable and responsible aquaculture practices.

## MATERIALS AND METHODS

### Ethical approval

All experimental procedures involving live prawns were reviewed and approved by the Animal Ethics Committee of Kalasin University, Thailand (Approval No. KSU-AE-036). The study was conducted in strict accordance with the Ethical Principles and Guidelines for the Use of Animals in Science (National Research Council of Thailand, 2015) and adhered to the Institutional Animal Care and Use Policy of Kalasin University.

Throughout the study, all procedures, sample collection, bacterial isolation, and laboratory analyses were performed with careful consideration to minimize stress, pain, and suffering to the animals. Prawns were handled humanely during collection and were not subjected to invasive or harmful treatments. After sample collection, all prawns were promptly returned to their rearing ponds under optimal environmental conditions.

The research was designed to comply with the “Three Rs” principles (Replacement, Reduction, and Refinement) by minimizing the number of animals used, employing non-invasive sampling methods, and refining procedures to ensure ethical standards of animal welfare.

### Study period and location

This study was conducted from April 2022 to November 2022 at the Animal Hospital Laboratory, Faculty of Agricultural Technology, Kalasin University, Thailand.

### Study area and sample collection

Samples were collected from six giant freshwater prawn farms located in Yang Talat District, Kalasin Province, Thailand (16°24′N, 103°31′E). Three rearing ponds were sampled from each farm, totaling 18 ponds. Soil samples were aseptically collected from the bottom sediments of the ponds. Six *Bacillus*-like isolates were initially screened for enzyme production and hemolytic safety. Based on preliminary evaluations, three isolates exhibiting the most promising probiotic traits, *B. pseudomycoides*, *B. safensis*, and *B. infantis*, were selected for detailed characterization.

### Isolation and identification of *Bacillus* strains

The bacterial isolates were identified based on partial sequencing of the *16S ribosomal RNA* gene. The obtained sequences were compared against those in the National Center for Biotechnology Information GenBank database using the Basic Local Alignment Search Tool algorithm and isolates with ≥98% sequence identity were considered correctly identified. The isolates *B. pseudomycoides* (GenBank accession no. MG02–0673), *B. safensis* (MG02–0676), and *B. infantis* (MG02–0677) were previously confirmed and preserved at −80°C in 20% (v/v) glycerol until further analysis.

### Inoculum preparation

Each *Bacillus* strain was reactivated by inoculating 0.1 mL of frozen culture into 4 mL of de Man, Rogosa, and Sharpe (MRS) broth (HiMedia, India) and incubating at 37°C for 24 h. A 10% inoculum from the overnight culture was subsequently transferred to 10 mL of fresh MRS broth (HiMedia) and incubated under the same conditions for an additional 24 h, until it reached the early stationary phase. The final bacterial concentration was adjusted to approximately 1 × 10^8^ colony-forming units/mL. The procedure was adapted from standard microbial cultivation protocols described by Michael and Pelczar [14].

### Evaluation of probiotic properties

#### Digestive enzyme activity assays

The ability of *B. pseudomycoides*, *B. safensis*, and *B. infantis* to produce key digestive enzymes, protease, amylase, and lipase, was evaluated using both semiquantitative and quantitative assays. The relative protease and amylase activities were expressed as the halo/colony (H/C) ratio, following the methodology described by Sam-On *et al*. [11] earlier for comparative *Bacillus* enzyme assessment. The quantitative lipase activity (U/mL) of *B. safensis* was determined using the method described by Larrondo *et al*. [15].

#### Hemolytic activity

The hemolytic activity of *B. pseudomycoides*, *B. safensis*, and *B. infantis* was determined by culturing each strain in MRS broth at 37°C for 24 h, followed by streaking onto Columbia agar plates (HiMedia) containing 5% defibrinated sheep blood. The plates were incubated at 37°C for 24 h, and hemolysis patterns were classified as follows:

- β-hemolysis: Complete lysis of red blood cells (RBC), forming a clear zone around the colony.
- α-hemolysis: Partial lysis, forming a greenish zone.
- γ-hemolysis: No visible change around colonies, indicating a non-hemolytic nature [16].

#### Antibiotic susceptibility testing

Antibiotic susceptibility of the isolates was assessed using the disk diffusion method, following the guidelines of the National Committee for Clinical Laboratory Standards (NCCLS) [17]. Antibiotic disks (Oxoid, England) were applied to nutrient agar plates inoculated with each *Bacillus* strain. Reference strains, *Staphylococcus aureus* ATCC 25923, *Neisseria gonorrhoeae* ATCC 49226, *Escherichia coli* ATCC 25922, and *Streptococcus pneumoniae* ATCC 49619, obtained from the TISTR Culture Collection, Thailand Institute of Scientific and Technological Research, Pathum Thani, Thailand, were used as controls to verify antibiotic efficacy. The diameters of inhibition zones (mm) were measured and interpreted as sensitive (S ≥ 21 mm), moderately susceptible (MS) (MS = 16–20 mm), or resistant (R ≤ 15 mm) for Gram-positive bacteria according to NCCLS criteria [17].

### Statistical analysis

All experiments were conducted in triplicate and independently repeated three times to ensure reproducibility. Data were expressed as mean ± standard deviation. Statistical analyses were performed using one-way analysis of variance followed by Duncan’s multiple range test to determine significant differences (p < 0.05). Positive controls included *B. subtilis* ATCC 6633 for enzyme activity and *S. aureus* ATCC 25923 for hemolytic testing, while sterile medium served as the negative control.

## RESULTS

### Digestive enzyme activity

The digestive enzyme activities of the three *Bacillus* isolates are summarized in Table 1. All strains exhibited detectable amylase and protease activities, with H/C ratios ranging from 1.75 ± 0.16 to 2.27 ± 0.11, whereas lipase activity was undetectable (0.00 ± 0.00 U/mL). Among the isolates, *B. safensis* demonstrated the highest amylase (2.27 ± 0.11) and protease (1.96 ± 0.14) activities, followed by *B. pseudomycoides* and *B. infantis*. An H/C ratio greater than 1.0 indicates strong extracellular enzyme secretion; therefore, these results confirm that all isolates exhibit significant amylolytic and proteolytic capabilities. Such enzymatic activity supports efficient starch and protein degradation, suggesting that these *Bacillus* strains could enhance feed digestion and nutrient assimilation in *M. rosenbergii* culture systems.

**Table 1:** Digestive enzyme activity of *Bacillus* isolates expressed as halo/colony (H/C) ratio and enzyme activity (U/mL).

*Bacillus* strains	Type of enzyme activity		

	Protease activity (H/C ratio ± SD)	Amylase activity (H/C ratio ± SD)	Lipase activity (U/mL ± SD)
*Bacillus pseudomycoides*	1.88 ± 0.15	2.10 ± 0.12	0.00 ± 0.00
*Bacillus safensis*	1.96 ± 0.14	2.27 ± 0.11	0.00 ± 0.00
*Bacillus infantis*	1.75 ± 0.16	2.05 ± 0.13	0.00 ± 0.00

The H/C ratio (halo/colony) represents the diameter of the clear zone relative to the colony size, with values >1.0 indicating significant extracellular enzyme secretion. Lipase activity is expressed in U/mL. All values are presented as mean ± standard deviation from triplicate determinations (n = 3). The results indicate that all *Bacillus* isolates exhibited amylase and protease activities but no detectable lipase activity.

### Hemolytic activity

**Figure 1:**
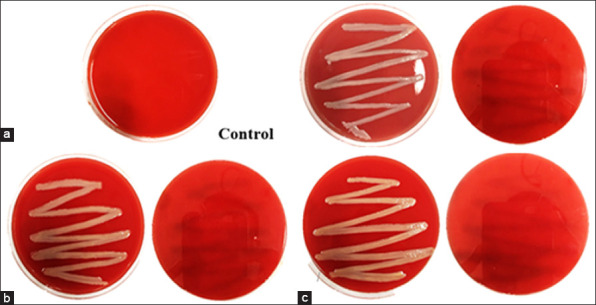
Hemolytic activity of *Bacillus* isolates on blood agar plates. Plates inoculated with (a) *Bacillus pseudomycoides*, (b) *Bacillus safensis*, and (c) *Bacillus infantis* show γ-hemolysis (no clear zone around colonies), indicating that they are non-hemolytic and safe for probiotic application. Note: γ-hemolysis (gamma-hemolysis) indicates no red blood cell lysis and is considered a non-pathogenic trait of probiotic candid.

The hemolytic properties of the three *Bacillus* isolates are shown in Table 2. All strains (*B. pseudomycoides*, *B. safensis*, and *B. infantis*) exhibited γ-hemolysis, indicating the absence of RBC lysis and confirming their non-hemolytic nature. No α- or β-hemolysis was observed, which implies that the isolates do not produce hemolysins or other cytotoxic metabolites associated with pathogenicity. These findings demonstrate that the isolates are safe for probiotic application, meeting one of the essential criteria for candidate probiotic bacteria in aquaculture (Figure 1).

**Table 2:** Hemolytic activity of the *Bacillus* isolates used in this study.

*Bacillus* strains	Hemolysis pattern		

	Alpha (α)	Beta (β)	Gamma (γ)
*Bacillus pseudomycoides*	-	-	+
*Bacillus safensis*	-	-	+
*Bacillus infantis*	-	-	+

α = Alpha-hemolysis (partial red blood cell lysis), β = Beta-hemolysis (complete lysis), γ = Gamma hemolysis (no lysis). All *Bacillus* isolates exhibited γ-hemolysis, indicating non-hemolytic and safe and suitable for probiotic application.

### Antibiotic susceptibility profile

**Figure 2:**
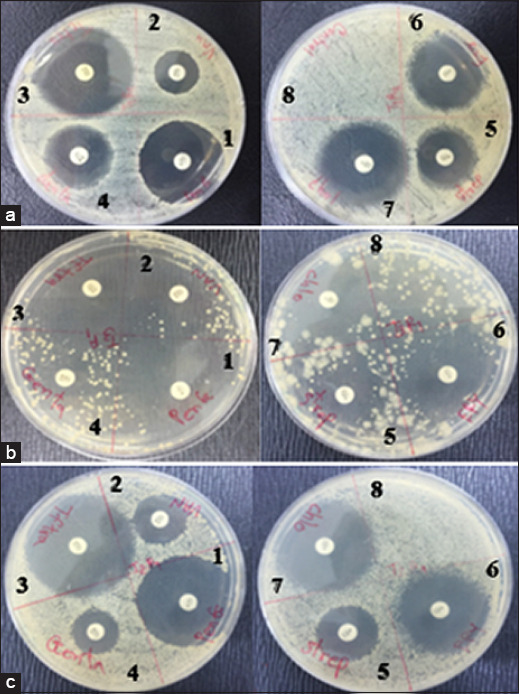
Antibiotic susceptibility assay of *Bacillus* isolates using disk diffusion. Plates inoculated with (a) *Bacillus pseudomycoides*, (b) *Bacillus safensis*, and (c) *Bacillus infantis* show inhibition zones produced by seven antibiotic disks: (1) penicillin G, (2) vancomycin, (3) tetracycline, (4) gentamicin, (5) streptomycin, (6) erythromycin, and (7) chloramphenicol; (8) represents the control disk. Note: Antibiotic susceptibility was evaluated based on the inhibition zone diameter (mm) according to the Clinical and Laboratory Standards Institute guidelines [18]. All isolates exhibited broad susceptibility to most antibiotics tested, with *Bacillus pseudomycoides* and *Bacillus safensis* showing moderate susceptibility to vancomycin.

The antibiotic susceptibility profiles of the isolates are presented in Table 3 and Figure 2. All *Bacillus* strains were susceptible to most of the tested antibiotics, including penicillin G, tetracycline, streptomycin, erythromycin, and chloramphenicol, with inhibition zones ranging from 25.37 ± 2.90 to 42.35 ± 3.50 mm. Moderate susceptibility to vancomycin was observed in *B. pseudomycoides* (18.19 ± 1.90 mm) and *B. safensis* (19.03 ± 1.80 mm), while *B. infantis* (20.23 ± 1.50 mm) remained within the susceptible range. Although minor variation occurred in the gentamicin response, all isolates were still classified as sensitive according to the Clinical and Laboratory Standards Institute interpretive criteria [18].

**Table 3:** Mean inhibition zone diameters (mm ± SD) of *Bacillus* isolates against selected antibiotics.

Antibiotic	Disk Potency	*Bacillus* spp.			Clinical interpretation (S/MS/R)

		*Bacillus pseudomycoides*	*Bacillus safensis*	*Bacillus infantis*	
Penicillin G	10 U	33.36 ± 2.80	36.12 ± 2.60	41.08 ± 3.00	Susceptible (S)
Vancomycin	30 µg	18.19 ± 1.90	19.03 ± 1.80	20.23 ± 1.50	Moderate susceptibility (MS)/S*
Tetracycline	30 µg	37.55 ± 3.10	39.52 ± 3.20	42.35 ± 3.50	Susceptible (S)
Gentamicin	10 µg	25.55 ± 2.50	17.35 ± 1.80	14.27 ± 1.70	Susceptible (S)
Streptomycin	10 µg	25.37 ± 2.90	21.15 ± 2.70	18.74 ± 2.40	Susceptible (S)
Erythromycin	15 µg	29.44 ± 2.60	29.89 ± 2.50	27.53 ± 2.30	Susceptible (S)
Chloramphenicol	30 µg	32.62 ± 3.00	33.49 ± 2.90	26.35 ± 2.50	Susceptible (S)

S = susceptible (organism is inhibited by the usual therapeutic concentration of the antibiotic); MS = Moderately susceptible (organisms are inhibited only at higher antibiotic concentrations); R = Resistant (organism not inhibited by the usual therapeutic concentration). Data are presented as the mean ± standard deviation (SD) from triplicate determinations (n = 3). Interpretations were based on the Clinical and Laboratory Standards Institute (CLSI, 2024) guidelines. All isolates showed broad susceptibility to most tested antibiotics, indicating a favorable safety profile for probiotic application.

These results indicate that the isolates exhibit a broad antibiotic-sensitive profile, reflecting low risk of antibiotic resistance transfer. This reinforces their biosafety and suitability as probiotic candidates for sustainable aquaculture practices.

## DISCUSSION

### Probiotic potential of *Bacillus* strains in freshwater prawn aquaculture

Recent research has identified the *Bacillus* genus as a promising source of probiotic bacteria in aquaculture, particularly for freshwater prawns (*M. rosenbergii*). The present study evaluated three *Bacillus* strains, *B. pseudomycoides*, *B. safensis*, and *B. infantis*, isolated from prawn culture systems and revealed several beneficial characteristics that support their suitability as probiotics.

All isolates demonstrated the ability to secrete digestive enzymes, including amylase and protease, which play key roles in starch and protein degradation. These activities are essential for enhancing nutrient assimilation, improving feed digestibility, and supporting overall growth performance in crustaceans. The strong amylolytic and proteolytic activities observed in our study are consistent with those reported by Wei *et al*. [19], who identified enzyme production as one of the most critical probiotic attributes. Similarly, *Bacillus* species have been shown to improve nutrient absorption and inhibit pathogenic bacteria such as *A. hydrophila* in prawns [11], further validating the functional probiotic potential of these isolates.

### Safety assessment and antibiotic susceptibility

Safety evaluation is a fundamental prerequisite for the use of probiotics in aquaculture. All three isolates exhibited γ-hemolysis, indicating the absence of hemolytic or cytotoxic activity and confirming their non-pathogenic nature [20]. Furthermore, the isolates were generally sensitive to a broad range of antibiotics, including penicillin G, tetracycline, streptomycin, erythromycin, and chloramphenicol.

While *B. pseudomycoides* and *B. safensis* showed moderate resistance to vancomycin, this behavior is considered intrinsic rather than plasmid-mediated. Intrinsic vancomycin resistance in certain *Bacillus* species is attributed to alterations in peptidoglycan precursors and does not involve transferable resistance genes [19, 21]. Therefore, the risk of antibiotic resistance dissemination in aquaculture environments is minimal. These findings align with previous studies by Meidonga *et al*. [21], which reported similar patterns of limited antibiotic resistance in *Bacillus aerius* B81e. Collectively, the overall antibiotic sensitivity profile of these isolates confirms their biosafety and suitability for probiotic application.

### Immunomodulatory and physiological benefits

Beyond digestion and safety, *Bacillus* probiotics are recognized for their immunomodulatory effects. Previous studies by Labaiden *et al*. [13] and Qiu *et al*. [22] have demonstrated that dietary supplementation with *Bacillus* species can enhance immune parameters, including leukocyte activity and phagocytosis, in prawns. Similar findings were reported by Labaiden *et al*. [23] for *B. licheniformis*, which stimulated immune response and improved resistance to infections in *M. rosenbergii*. The enzyme-secreting capacity and safe profile of the isolates in this study indicate their potential to promote immune health in addition to improving digestion, thereby contributing to enhanced resilience against pathogenic challenges.

### Comparison with commercial probiotics

When compared with commonly used commercial probiotics such as *B. subtilis* and *B. licheniformis*, the isolates identified in this study exhibited comparable protease activity but higher amylase activity, suggesting greater potential for carbohydrate digestion. The enhanced enzymatic efficiency could translate into improved feed conversion and nutrient utilization. Ding *et al*. [24] have shown that *Bacillus*-derived enzymes can increase feed conversion efficiency in crustaceans by up to 20%. Furthermore, the native isolates displayed greater environmental tolerance, likely due to adaptation to local pond conditions in Kalasin Province. This trait offers a practical advantage for their integration into tropical aquaculture systems, where water quality fluctuations are common.

### Applied prospects and future work

From an application standpoint, the identified isolates could be developed as feed- or water-based probiotics to enhance digestion, immunity, and disease resistance in *M. rosenbergii* culture. Encapsulation and biofloc-based delivery methods may further improve their stability and efficacy during field application. The current findings provide valuable baseline data for the development of regionally adapted *Bacillus* probiotics. However, further *in vivo* trials are essential to validate their efficacy under commercial farming conditions and to evaluate their effects on growth performance, survival, and immune biomarkers.

## CONCLUSION

The present study demonstrated that three *Bacillus* strains, *B. pseudomycoides*, *B. safensis*, and *B. infantis*, isolated from giant freshwater prawn (*M. rosenbergii*) farms in Kalasin Province, Thailand, possess key probiotic characteristics. All strains exhibited strong amylase and protease activities (H/C ratios 1.75–2.27) but lacked lipase activity, indicating their capability to enhance carbohydrate and protein digestion in prawns. The isolates were non-hemolytic (γ-hemolysis), confirming their safety, and showed a broad antibiotic-sensitive profile, with only moderate intrinsic resistance to vancomycin. These findings confirm their functional potential as safe and efficient probiotics for aquaculture applications.

From a practical perspective, the use of these native *Bacillus* strains could improve feed utilization, growth performance, and immune resilience in *M. rosenbergii* culture while reducing dependency on antibiotics and mitigating environmental contamination. The major strength of this study lies in the use of locally adapted strains isolated from tropical aquaculture environments, which likely enhances their environmental tolerance and long-term stability.

However, the study was limited to *in vitro* evaluations; therefore, *in vivo* validation under commercial farming conditions is essential. Future research should focus on assessing growth performance, immune modulation, pathogen inhibition, and molecular safety profiles.

In conclusion, these native *Bacillus* isolates represent promising candidates for sustainable probiotic development, aligning with One Health and environmentally responsible aquaculture principles.

## DATA AVAILABILITY

The supplementary data can be made available from the corresponding author upon request.

## AUTHORS’ CONTRIBUTIONS

KP, NK, PK, and TB: Planned the study, screening process, data analysis, and drafted the manuscript. UK: Data analysis and interpretation of results. KP and TB: Interpretation of results and revision of the manuscript. All authors have read and approved the final version of the manuscript.
